# Effect of dietary biochar supplementation on growth, blood constituents, digestive enzymes, intestinal microbiota and viscosity, antioxidant indices, and carcass traits of broiler chicks

**DOI:** 10.1007/s11250-025-04754-4

**Published:** 2026-01-05

**Authors:** Ahmed M. Emam, Shaaban Saad Elnesr, Bothaina Y. Mahmoud, Ensaf A. El-Full, Ibrahim A. Abdel-Kader, Doaa A. Semida

**Affiliations:** Department of Poultry Production, Faculty of Agriculture, Fayoum University, Fayoum, 63514 Egypt

**Keywords:** Biochar, Growth, Blood, Intestine, Broiler

## Abstract

This study examined the effects of incorporating biochar into broiler diets on productive performance and physiological status. In total, 240 unsexed one-day-old broiler chicks were divided into three treatments, each with four replicates of 20 chicks. The first group received a basal diet without biochar (control). The second and third treatments received the basal diet supplemented with 1.5% and 3% biochar, respectively. The results revealed that biochar supplementation significantly improved growth, feed efficiency, and carcass characteristics. Biochar supplementation had a significant impact on blood biochemistry, reducing levels of aspartate aminotransferase, uric acid, creatinine, and total bilirubin, while increasing total protein, globulin, calcium, and phosphorus levels. Hematological parameters remained mostly stable, except for an increase in hematocrit and serum iron levels, along with a reduction in total iron-binding capacity. Biochar notably improved antioxidant indices in both serum and liver. Digestive enzyme activities, particularly amylase activity, were significantly enhanced by biochar. Furthermore, biochar had a positive effect on caecal microbiota, increasing beneficial bacteria and reducing harmful bacteria (*p* < 0.001). Intestinal pH was increased and viscosity was decreased with higher biochar levels. In summary, adding biochar at levels up to 3% to broiler diets had beneficial impacts on various aspects of health, performance, intestinal microbiota, and blood constituents.

## Introduction

Poultry production plays a vital role in ensuring a sustainable source of animal protein thereby contributing to community self-sufficiency. There is a growing need for effective alternatives that can support poultry health and performance without the adverse effects associated with antibiotic use (Elgeddawy et al. 2020; Rafeeq et al. 2022). Generally, the increasedinterest in natural sources is associated with greaterbenefits and fewer health concerns. Currently, there is an essential need for feed supplements that demonstrate superior sustainability attributes, which can be safely incorporated into diets to promote poultry health and productivity. One promising option identified as a feed supplement is biochar (Gerlach and Schmidt 2012; Dim et al. 2022). Biochar is a carbon-rich porous material produced by partial oxidation of carbonaceous organic sources such as wood and plants in a process known as pyrolysis, which involves heating the biomass in limited-oxygen conditions (Bhattacharya et al. 2024). The production cost of biochar can vary widely, depending on factors such as feedstock availability, pyrolysis technology, and production scale (Patel and Panwar 2024). The use of biochar represents a promising opportunity to improve the sustainability of poultry production while meeting the increasing global demand for animal-derived food. Its availability, cost-effectiveness, and nutritional advantages make biochar a compelling option to consider. Biochar has an extremely high surface area and porosity combined with low density; which provides excellent adsorptive properties that enable it to bind pollutants and reduce harmful gases (Tan et al. 2016; Al-Khalaifah and Al-Nasser 2023). Depending on the feed material and pyrolysis conditions, biochar typically contains 38.98–80% carbon, 0.08–0.8% nitrogen, 5.06% hydrogen, and 4.47% ash (Willson et al. 2019; Sharma and Mohanty 2021; El-Ghalid et al. 2022). Owing to the characteristics of this feed additive and its negatively charged ionic state, biochar’s beneficial effects are likely related to its adsorptive capabilities which may exert a therapeutic effect on the gastrointestinal tract (Kutlu et al. 2001). However, the precise mechanisms underlying these beneficial effects remain to be clearly delineated.

Biochar has been shown to enhance growth performance, improve blood characteristics, reduce fecal microbial shedding, and enhance gut health and feed efficiency in poultry (Willson et al. 2019). Biochar supplementation in broiler diets enhances digestion, feed efficiency, and energy absorption while also binding toxins like dioxin, glyphosate, and mycotoxins. This, in turn, helps protect the digestive system and improves intestinal microbiota. Studies have shown that biochar inclusion levels ranging from 0.2% to 6% can deactivate digestive toxins, enhance intestinal microbiota, reduce mortality, and improve growth performance (Gerlach and Schmidt 2012; Kutlu et al. 2001; Dim et al. 2022). However, higher levels of 7% or above have been reported tonegatively impact growth and final weight gain (Odunsi et al. 2007). El-Ghalid et al. (2022) clarified that the inclusion of biochar in broiler diet (1, 2, 4, and 6%) enhanced the growth performance of chicks without exertingany harmful influences on liver and kidney functions, antioxidant indices, and lipid profile.

Previous investigations have demonstrated that using biochar as a feed supplement in animal feed has been correlated with affirmative results, such as better digestion, feed conversion ratio (FCR), body weight gain (BWG), as well as mitigation of greenhouse gas emission mitigation, and protective effects against intoxication and viral or bacterial diseases (Kalus et al. 2019; Kammann et al. 2017). Furthermore, using biochar as a feed additive in broiler diets enhanced performance via improving the FCR and BWG (Kutlu et al. 2001). Moreover, the inclusion of biochar in poultry diets can improve the general health of the birds as it reduces pathogenic bacteria (Prasai et al. 2016), and removes harmful metabolites in the digestive tract (Gerlach and Schmidt 2012). From these observations, using biochar as a feed additive for poultry emerges as a promising solution, as it can be used for improving both the productive performance and overall health of birds.

It is hypothesized that the inclusion of biochar in the diet as a feed additive may exert favorable influences on broiler chicks. Although the inclusion of biochar in feed has demonstrated significant advantages in poultry production, existing research remains fragmented and insufficient. Many studies have overlooked important factors, including digestive enzymes, iron profile, blood ammonia, and intestinal viscosity. Furthermore, differences in the source and inclusion levels of biochar have resulted in inconsistentoutcomes and hindered the development of standardized recommendations. Therefore, the aim of the current study was to assess the influences of dietary biochar supplementation as a functional feed additive on growth performance, feed efficiency, some blood constituents, iron profile, blood ammonia, digestive enzymes, intestinal microbiota and viscosity, antioxidant indices, and carcass traits of broiler chicks.

## Materials and methods

### Experimental design, birds and diets

A total of 240 unsexed one-day-old broiler chicks (Arbor Acres) at were randomly distributed at equal body weights (46.33 g ± 0.15 g) into three groups: the control and two treatments, which consisted of the control diet plus 1.5% and 3% biochar as a feed additive, respectively. Each group consisted of four replicates of 20 chicks each. Biochar used in this study was produced through the incomplete pyrolysis of mango tree branches or wood, at approximately 550 °C under oxygen-limited conditions. Chicks were reared on floor pens with deep straw litter, with 20 chicks each throughout the experimental period. The ambient temperature was maintained at 32 °C from 0 to 7 days and gradually decreased to 18 °C by day 35 of age.

Three maize-soybean meal-basad diets (starter 1–10 days, grower 11–24 days, and finisher 25–35 days) were formulated to meet the recommended nutrient requirements for broilers according to the broiler management guidelines (Aviagen 2022). Table 1 exhibited the ingredients and chemical composition of the basal diets. Feed in mash form and water were provided *ad libitum*.

**Table 1:** Ingredients and chemical composition of the basal diets

Ingredients	Starter diet	Grower diet	Finisher diet
	From 1: 10 days of age	From 11: 24 days of age	From 25: 35 days of age
Yellow corn	60.40	65.00	69.50
Soybean meal (46% CP)	25.00	25.00	14.15
Corn gluten	9.64	5.70	10.00
Di-calcium phosphate	2.04	1.65	1.65
CaCO3	1.20	1.00	1.00
NaCl	0.30	0.30	0.30
Vitamin and mineral premix^1^	0.30	0.30	0.30
Lysine	0.51	0.50	0.56
Methionine	0.16	0.15	0.14
Soybean oil	0.45	0.40	2.40
Total	100.00	100.00	100.00
**Calculated analysis**			
Crude protein (CP) %	22.97	20.98	18.99
ME (kcal/kg diet)	2998	3002	3209
Ether extract%	3.19	3.21	5.40
Crude fiber%	3.27	3.29	2.72
Calcium%	0.99	0.83	0.80
Available Phosphorus %	0.50	0.43	0.42
Methionine%	0.55	0.51	0.49
Lysine *%*	1.33	1.29	1.10

^1^ Each 3.0 Kg of the broiler Vit. and Min. premix contains: Vit. A 12,000,000 IU, Vit. D_3_ 2,000,000 IU, Vit. E 40,000 mg, Vit. K_3_ 4000 mg, Vit. B_1_ 3000 mg, Vit. B_2_ 6000 mg, Vit. B_6_ 4000 mg, Vit. B_12_ 30 mg, choline chloride 350,000 mg, biotin 80 mg, folic acid 1500 mg, nicotinic acid 30 g, niacin 30,000 mg, pantothenate acid 12,000 mg, Zn 70,000 mg, Cu 4 g, Fe 40,000 mg, Co 10,000 mg, Se 200 mg, I 300 mg, Mn 70,000 mg and completed to 3.0 Kg by calcium carbonate

ME: Metabolizable energy

### Growth performance

Live body weight (BW) of chicks was individually measured and feed intake (FI) per pen was recorded, BWG was calculated as the difference between final BW and initial BW, and FCR was also calculated. The European production efficiency factor (EPEF) at day 35 was calculated as follows: EPEF = [final BW × survival rate%/FCR×35] × 10.

The growth rate (GR_1 − 35_%) was calculated using the following formula:

GR_1 − 35_= [(W_35_-W_1_)×100]/[ 0.5 (W_35_ + W_1_)]

### Carcass traits

At the end of the experiment (35 days of age), eight birds from each treatment group were reweighed and slaughtered by cutting the jugular vein, then defeathered and eviscerated. All carcass traits (carcass%, gizzard%, liver%, heart%, thigh bone%, and breast bone%) were calculated relative to LBW.

### Measurements of digesta viscosity and pH

At 35 days of age, the entire gastrointestinal tract was immediately removed for visual evaluation and digesta sampling. Digesta samples from the duodenum, jejunum, ileum, and cecum were separately collected into separate tubes and vortexed to obtain a homogenous mixture from each intestinal segmentfor every bird. The pH of the homogenized contents of the duodenum, jejunum, ileum, and cecum samples was measured using a digital pH meter (pH 315i, WTW Wissenschaftlich- TechnischeWerkstätten, Weilheim, Germany) immediately after sampling following a 1:10 dilution with saline solution. The viscosity of intestinal digesta was determined according to Tsiouris et al. (2013) using a Brookfield DV-II + PRO Digital Viscometer (Brookfield Engineering Laboratories, Stoughton, MA, USA). Two readings were taken from each sample and expressed in in units of centipoise.

### Caecal microbiota analysis

Samples (8/treatment) were collected from both caeca of each bird within each group. Total anaerobes, *Escherichia coli*, and *Lactobacillus* counts were enumerated respectively using duplicate plate count agar, Mac Conkey agar, and MRS agar (Tuohy et al. 2002). The plates were incubated for 24 to 72 h at 37 °C, and then colony numbers were counted. The findings were expressed as log10 colony-forming units per gram of digested content of the intestine.

### Biochemical analyses

At the end of the experiment (35 days of age), blood sampls were collected from the slaughtered chicks (8 chicks/treatment) by cutting the jugular vein. The blood sample/chick was collected into two tubes. The 1st tube contained whole blood with an anticoagulant to determine haematological parameters (hemoglobin (Hb), hematocrit, red blood cells (RBCs), and white blood cells (WBCs). The 2nd tube (without an anticoagulant) was centrifuged for 15 min (755 g value) to separate the serum that was stored at − 20◦C until the laboratory analysis.

### Antioxidant indices

Total antioxidant capacity (T-AOC) and superoxide dismutase (SOD) in the serum and liver using commercial kits were analyzed colorimetrically using an infinite F50 spectrophotometer (Tecan Trading AG, Männedorf, Switzerland). For liver analysis, samples were prepared and homogenized according to the manufacturer’s instructions. Catalogue numbers of the kits used: (T-AOC, E-BC-K136-M and SOD E-BC-K019-S Elabscience -United States).

### Blood biochemical parameters

Serum biochemical parameters (albumin, total protein, creatinine, uric acid, total iron-binding capacity (TIBC), iron, aspartate aminotransferase (AST), alanine aminotransferase (ALT), ammonia, and total bilirubin levels) were determined colorimetrically using appropriate commercial diagnostic kits produced by Abcam Inc (UK), Cat. No. (Total protein-219272, Albumin- ab235628, Uric Acid- ab65344, TIBC- ab239715, ALT- ab241035, AST- ab105135, Ammonia- ab102509, Creatinine- ab204537 and Total Bilirubin- ab235627). Lipase and amylase enzymes were assayed according to Friedman and Young (2005), and trypsin enzyme was determined using the Bovine Trypsin ELISA Kit MBS706461.

### Statistical analysis

The recorded data (growth, carcass, and biochemical parameters) were statistically analyzed using a one-way analysis of variance (ANOVA) using SPSS software (v.22.0) to calculate the treatment-specific means using the following model :

$${Y_{ij}} = {\text{ }}\mu {\text{ }} + {\text{ }}{B_i} + {\text{ }}{e_{ij}}$$

Where: Y_ij_ = Observed measurement. µ = Overall mean. B_i_ = Effect of biochar treatment, e_ij_ = Experimental error. Means of treatments were compared using Duncan’s multiple range test. The data were analyzed to examine whether the significant trends were linear or quadratic. A level of *p* < 0.05 was used as the criterion for statistical significance. All data were displayed as mean ± standard error.

## Results

### Growth performance

**Fig. 1:**
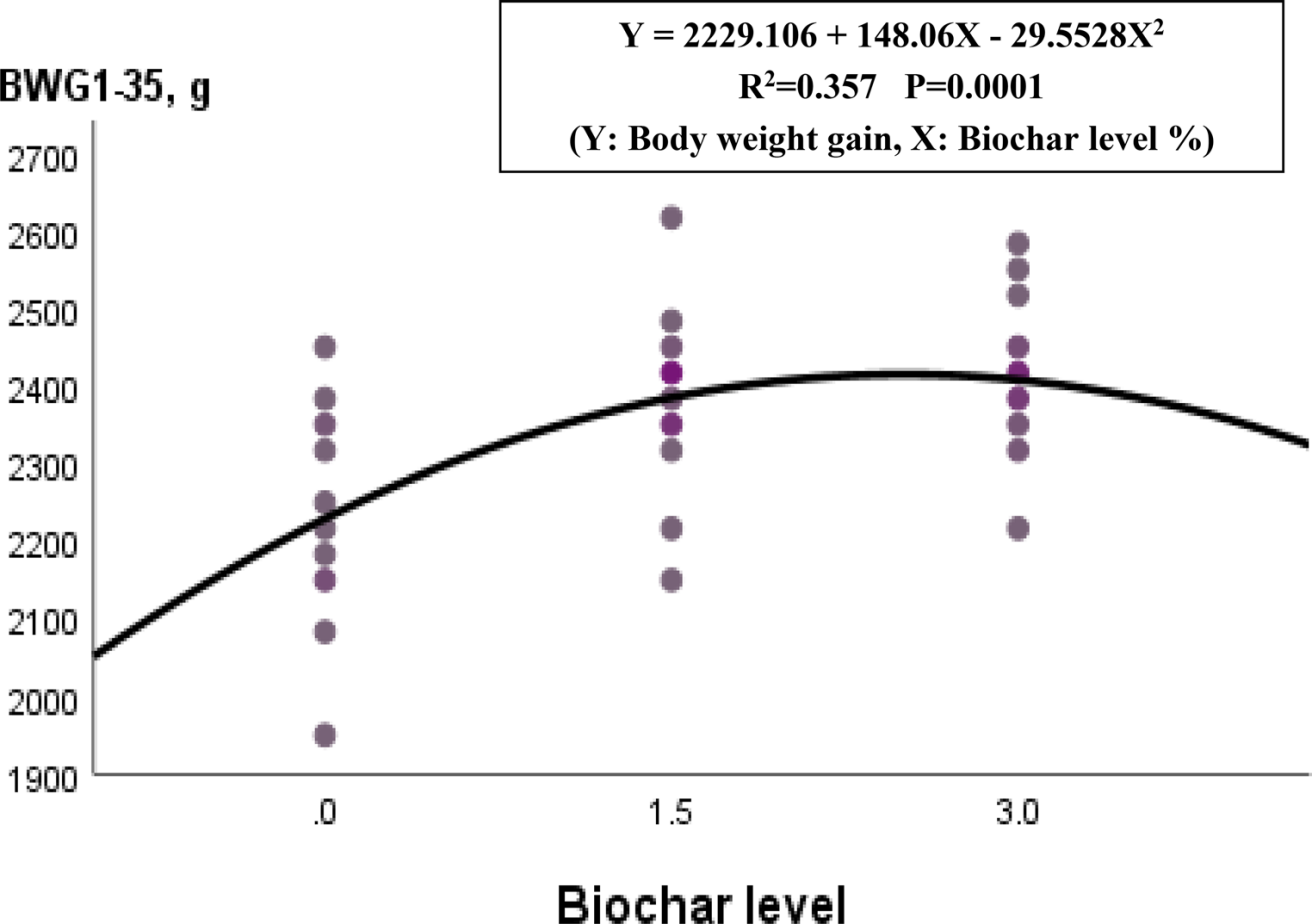
Polynomial regression analysis between BWG1-35 and dietary biochar levels of broiler diets

The influences of dietary treatment on LBW_1_ (live body weight at 1 day of age), LBW_1 − 35_ (live body weight at 35 days of age), BWG_1 − 35_ (body weight gain from 1 to 35 days of age), GR_1 − 35_% (growth rate during 1–35 days of age), FI_1 − 35_ (feed intake during 1–35 days of age), FCR_1 − 35_ (feed conversion ratio during 1–35 days of age), and EPEF (European production efficiency factor at day 35) are shown in Table 2. Except for LBW_1_, the inclusion of biochar in broiler diets significantly improved (*p* < 0.0001) all studied growth traits. Chicks fed diets containing 3% biochar followed by those fed 1.5% biochar exhibited significantly heavier LBW_35_, higher BWG_1 − 35_, GR_1 − 35_%, along with lower FI_1 − 35_ and better FCR_1 − 35_ compared with the control group. The 3% biochar group insignificantly surpassed the 1.5% group by 100.94, 100.95, 100.03, 99.54, and 98.51 for each of LBW_35_, LBG_1 − 35_, GR_1 − 35_%, FI_1 − 35_ and FCR, respectively. Regarding the EPEF, broilers fed diets supplemented with 3% and 1.5% biochar exceeded their control by 22.32% and 19.45%, respectively, while the 3% biochar group surpassed the 1.5% group by 2.41%. The results of the polynomial regression statistical analysis demonstrated that the optimal range of biochar supplementation in broiler diets was between 1.5 and 3%, with the highest performance growth was observed at 3% (Fig. 1).

**Table 2:** Effect of Biochar treatment on productive performance of broiler chicks

Items	LBW_1_	LBW_35_	BWG_1 − 35_	GR_1 − 35_%	FI_1 − 35_	FCR_1 − 35_	EPEF
Control	46.26	2275.38^b^	2229.11^b^	192.00^b^	3329.52^a^	1.50^a^	427.95
Biochar 1.5%	46.33	2431.04^a^	2384.71^a^	192.51^a^	3182.03^b^	1.34^b^	511.17
Biochar 3.0%	46.39	2453.83^a^	2407.43^a^	192.57^a^	3167.31^c^	1.32^b^	523.47
SEM	0.13	10.76	10.77	0.03	4.60	0.01	4.41
Probability	0.8086	0.0001	0.0001	0.0001	0.0001	0.0001	0.0001

^a, b,c^: Means in the same column followed by different letters are significantly different at (*p* ≤ 0.05);

LBW1: Live body weight at 1 day of age; LBW35: Live body weight at 35 days of age; BWG1-35: Body weight gain from 1 to 35 days of age; GR1-35%: Growth rate during 1–35 days of age; FI1-35: Feed intake during 1–35 days of age; FCR1-35: Feed conversion ratio during 1–35 days of age; EPEF: European production efficiency factor at day 35; SEM : Standard error of mean

### Blood biochemical parameters

As shown in Table 3, dietary supplementation of biochar at two levels (1.5 and 3%) significantly decreased values of serum AST, uric acid and creatinine compared with the control. The group fed a diet containing the higher level of biochar (3%) had lower blood ammonia than the control and the 1.5% biochar group. Chicks fed diets supplemented with 1.5% biochar had higher total protein and globulin levels than those fed 3% biochar or the control diet. In addition, biochar-supplemented groups had lower total bilirubin levels (*p* = 0.0001) than the control group. Blood calcium and phosphorus levels were significantly augmented (*p* < 0.01) in biochar-supplemented birds compared with the control group (Table 3).

**Table 3:** Effect of Biochar treatment on blood biochemical parameters

Items	Blood amonia (mmol/L)	ALT (U/l)	AST (U/l)	Uric acid (mg/dl)	Creatinine (mg/dl)	Total protein (g/dl)	Albumin (g/dl)	Globulin (g/dl)	Total bilirubin level (mg/dl)	Calcium (mg/dl)	Phosphorus (mg/dl)
Control	0.28^a^	6.00	191.08^a^	4.91^a^	0.43^a^	3.21^c^	1.16^c^	2.03^c^	0.97^a^	9.68^b^	7.51^b^
Biochar 1.5%	0.26^a^	5.91	185.01^b^	4.33^b^	0.40^b^	3.86^a^	1.41^b^	2.45^a^	0.76^b^	9.92^a^	7.73^a^
Biochar 3.0%	0.22^b^	5.83	183.35^b^	4.33^b^	0.38^b^	3.77^b^	1.54^a^	2.23^b^	0.77^b^	9.95^a^	7.89^a^
SEM	0.01	0.10	1.71	0.06	0.01	0.03	0.03	0.03	0.02	0.06	0.08
Probability	0.0001	0.2369	0.0148	0.0001	0.0013	0.0001	0.0001	0.0001	0.0001	0.0034	0.0055

^a, b,c^: Means in the same column followed by different letters are significantly different at (*p* ≤ 0.05); SEM : Standard error of mean; AST: Aspartate aminotransferase; ALT: Alanine aminotransferase

### Blood hematology and iron profile

The influence of different dietary biochar levels on the hematological parameters of broiler chicks is presented in Table 4. Adding biochar to the diet had no a significant impact (*p* > 0.05) on Hb, RBCs, and WBCs. However, increasing levels of dietary biochar supplementation significantly increased both HCT% and serum iron (*p* < 0.05), while markedly reducing values of TIBC (*p* = 0.0001).

**Table 4:** Effect of Biochar treatment on hematological parameters and iron profile

Items	Hemoglobin (g/dl)	Red blood cell (10^6^/mm^3^)	White blood cell (10^3^/mm^3^)	Hematocrit (%)	Serum iron (mg/dl)	Serum TIBC (mg/l)
Control	11.29	3.61	24.50	40.40^b^	0.27^b^	0.47^a^
Biochar 1.5%	11.30	3.62	24.79	41.66^a^	0.28^b^	0.42^b^
Biochar 3.0%	11.66	3.69	24.86	42.40^a^	0.29^a^	0.40^b^
SEM	0.18	0.08	0.21	0.35	0.01	0.01
Probability	0.2641	0.7326	0.5140	0.0025	0.0467	0.0001

^a, b,c^: Means in the same column followed by different letters are significantly different at (*p* ≤ 0.05); SEM : Standard error of mean; TIBC: total iron-binding capacity

### Antioxidant indices

The results of antioxidant indices in serum and liver, which serve as indicators for assessing oxidative stress, are presented in Table 5. Broilers fed diets supplemented with 1.5% and 3% biochar had significantly higher levels of T-AOC in serum and liver than those fed the control diet. Birds fed diets supplemented with 1.5% biochar had significantly higher levels of liver SOD than those fed either 3% biochar or the control diets, but biochar supplementation had no significant effect on serum SOD activity.

**Table 5:** Effect of Biochar treatment on antioxidant indices and digestive enzymes

Items	Serum		Liver		Amylase U/l	Lipase U/l	Trypsin U/l
	T-AOC (U/ml)	SOD (U/ml)	T-AOC (U/mg/ protein)	SOD (U/mg/protein)			
Control	12.03^b^	169.88	6.85^b^	163.50^c^	355.66^b^	22.30	26.35
Biochar 1.5%	13.72^a^	171.77	7.45^a^	170.45^a^	381.17^a^	22.33	26.90
Biochar 3.0%	13.69^a^	171.47	7.35^a^	168.82^b^	387.50^a^	23.33	27.25
SEM	0.18	0.90	0.11	0.41	3.65	0.21	0.51
Probability	0.0001	0.3474	0.0015	0.0001	0.0001	0.1509	0.4909

^a, b,c^: Means in the same column followed by different letters are significantly different at (*p* ≤ 0.05); SEM : Standard error of mean; T-AOC; Total antioxidant capacity; SOD: Superoxide dismutase

### Digestive enzymes

The results of digestive enzyme activities are summarized in Table 5. Results indicated that the inclusion of different levels of biochar significantly enhanced amylase activity (*p* = 0.0001) but did not affect lipase and trypsin activities (*p* > 0.05). The highest level of amylase activity was recorded in the group receiving 3% biochar, followed by the group receiving 1.5% biochar in the diet, both of which significantly exceeded the control group.

### Carcass characteristics

Carcass characteristics as affected by the biochar inclusion in broiler diets are presented in Table 6. The results indicated that diets containing 1.5% biochar supplementation resulted in significantly higher carcass% and lower heart%, whereas 3% biochar resulted in higher gizzard% compared with the control diet. No significant effects (*p* > 0.05) were observed for dietary biochar on liver%, thigh bone%, and breast bone%.

**Table 6:** Effect of Biochar treatment on carcass characteristics

Items	Carcass%	Gizzard%	Liver%	Heart%	Thigh bone%	Breast bone%
Control	72.84^b^	2.27^b^	2.79	0.76^a^	10.57	13.33
Biochar 1.5%	75.89^a^	2.33^b^	2.70	0.70^b^	10.79	12.88
Biochar 3.0%	74.89^a^	2.57^a^	2.76	0.74^ab^	10.33	12.86
SEM	0.62	0.06	0.06	0.02	0.45	0.69
Probability	0.0103	0.0001	0.5459	0.0187	0.7623	0.8805

^a, b^: Means in the same column followed by different letters are significantly different at (*p* ≤ 0.05); SEM : Standard error of mean

### Intestinal microbiota

The caecal microbiota, which are presented in Table 7, explained that the biochar supplementation had a significant preferable impact on the caecal microbiota by increasing the counts of favorable bacteria such as *Lactobacillus* and decreasing the counts of both *E. coli* and total anaerobes.

**Table 7:** Effect of Biochar treatment on intestinal ph and viscosity, and caecal microflora counts

Items	pH				Viscosity			Caecal microflora counts		
	Duodenum	Jejunum	Ileum	Cecum	Duodenum	Jejunum	Ileum	Lactobacillus sp	E. coli	Total anaerobes count
Control	5.03^b^	5.53^c^	6.38^c^	6.73^c^	2.57^a^	3.08^a^	3.85^a^	8.09^c^	7.27^a^	9.23^a^
Biochar 1.5%	5.39^a^	5.97^b^	6.72^b^	7.28^b^	2.05^b^	2.74^b^	3.06^b^	8.34^b^	6.26^b^	8.46^b^
Biochar 3.0%	5.51^a^	6.63^a^	7.29^a^	7.52^a^	1.93^c^	2.28^c^	2.71^c^	8.72^a^	6.25^b^	7.84^c^
SEM	0.05	0.07	0.10	0.08	0.04	0.06	0.06	0.10	0.11	0.09
Probability	0.0001	0.0001	0.0001	0.0001	0.0001	0.0001	0.0001	0.0008	0.0001	0.0001

^a, b,c^: Means in the same column followed by different letters are significantly different at (*p* ≤ 0.05); SEM : Standard error of mean

### Intestinal pH and viscosity

Intestinal pH and viscosity were significantly (*p* < 0.0001) affected by biochar inclusion in broiler diets. As biochar levels increased from 0 to 3%, the intestinal pH tended to increase, whereas viscosity tended to decrease in all examined intestinal parts, as shown in Table 7.

## Discussion

Over time, poultry producers will need to avoid the use of pharmaceutical or chemical feed additives, shifting instead toward natural alternatives (Abdel-Kader et al. 2025). Recently, biochar has attracted increasing attention as a sustainable and eco-friendly solution for improving the health status and feeding practices of poultry. In the present study, dietary supplementation with biochar at inclusion levels of 1.5% and 3% significantly enhanced the growth performance of birds. Similar findings were elucidated by Dim et al. (2018), Goiri et al. (2021), Al-Jumaily and Al-Jumaily (2022), and El-Ghalid et al. (2022). This improvement can be attributed to the adaptation of intestinal endothelial cell walls to the biochar inclusion, leading to enhanced villus development and improved absorptive function (Kutlu et al. 2001). Moreover, the addition of dietary biochar has enhanced the bioavailability of digested diet materials; through its ability to reduce surface tension by absorbing water, a process facilitated by the minerals present within it (Jiya et al. 2014; Rafiu et al. 2014). Consequently, this promotes an increased capacity for nutrient exchange within the intestine, slows the passage of digesta through the gastrointestinal tract, and ultimately enhances nutrient digestion and absorption (Schmidt et al. 2015). Majewska et al. (2011) reported that the utilization of 0.3% hardwood biochar improved BWG, feed efficiency, while significantly reduced mortality rates. These effects were attributed to the reduction of surface tension of the digestive pulp, detoxification of feed components, and mitigation of hepatic lipidosis. Dietary biochar improved FCR by altering the microbiota composition of the digestive tract (Prasai et al. 2016). Through its enhancement of the intestinal microbiota, biochar facilitates better digestion and enhances energy utilization, thereby leading to improved growth (Dim et al. 2018; Goiri et al. 2021; Al-Jumaily and Al-Jumaily 2022; El-Ghalid et al. 2022). Biochar supplementation demonstrates a significant positive influence on the overall health status of birds, as evidenced by the improved EPEF. Similarly, Goiri et al. (2021) and El-Ghalid et al. (2022), reported remarkable effects of dietary biochar on the EPEF, which can be attributed to biochar’s strong adsorptive capacity toward various toxins and pathogens. These benefits contribute to the promotion of growth, leading to considering biochar as a valuable nutritional supplement for poultry.

Dietary supplementation of biochar led to a significant reduction in blood ammonia, uric acid, and creatinine levels in broilers attributable to biochar’s adsorptive capacity for various gases, drugs, and fat-soluble substances (Kutlu et al. 2001). The decrease in blood ammonia level can positively contribute to improving the status of respiratory system.

Serum albumin, globulin, total protein, and total bilirubin serve as vital biomarkers used for assessing hepatic function and integrity. The significant reduction in serum total bilirubin levels observed in birds fed diets with 1.5% and 3% biochar suggests improved liver function. This effect decrease can be attributed to biochar’s ability to enhance liver detoxification, improve gut health, and potentially provide antioxidant protection. Additionally, biochar may influence bile acid metabolism, thereby affecting bilirubin processing and excretion. Conversely, dietary supplementation of biochar led to a significant increase in albumin, globulin, and total protein levels in broilers. These findings are consistent with those found by El-Ghalid et al. (2022), who reported that supplementation of biochar into the diets of broilers up to 6% can enhance serum biochemical profiles. The observed decreased levels of uric acid and creatinine indicate that the application of bird-feed biochar was beneficial for improving kidney function.

Previous investigations have elucidated the favorable effects of biochar supplementation in reducing AST and ALT concentrations in birds (Jiya et al. 2014; Dim et al. 2018, 2022). El-Ghalid et al. (2022) reported that broiler chicks fed biochar at levels from 1% up to 8% showed no significant changes in ALT and AST activities. However, birds fed diets containing activated coconut shell charcoal had significantly higher AST levels compared to the control group (Jiya et al. 2014).

In the present study, birds fed dietary biochar showed significantly higher levels of calcium and phosphorus compared to the control group. The inclusion of higher levels of biochar seems to reduce fecal phosphorus levels, potentially due to its ability to bind phosphorus (Cheron 2017). Consequently, this binding ability of biochar to both phosphorus and calcium, biochar-fed birds showed high levels of serum phosphorus and calcium.

The observed hematological parameters (RBCs, WBCs, and Hb) demonstrated numerical increases with increasing biochar addition to the diet. This shows that biochar is safe and does not negatively impact hematological function. This stability of these parameters implies that biochar does not interfere with blood cell production, disrupt physiological balance, or cause stress. El-Ghalid et al. (2022) reported that dietary biochar significantly increased RBCs, WBCs, and Hb levels in broilers. Consistently, Odunsi et al. (2007), and Kana et al. (2014) also indicated that dietary supplementation with biochar did not significantly influence the hematological parameters of broilers. Dim et al. (2018) reported that the birds fed dietary biochar showed improved utilization of the vitamin-mineral premix in the diet, particularly iron and B-complex vitamins. This enhancement may be attributed to the potential ability of biochar to bind anti-nutritional factors present in the birds’ gut, which might otherwise interfere with the utilization of these vitamins and minerals necessary for producing cellular components in the blood. The improved health status of the birds could be linked to the mitigation of physiological stress (as achieved by the use of zeolite, Emam et al. 2023). Finally, biochar as a feed additive can boost serum iron, which is an essential component of Hb, the oxygen-carrying protein in RBCs (Elnesr et al. 2022).

The results of the effect of biochar on antioxidants in the current study were consistent with Dim et al. (2022), who illuminated that there was an increase in serum antioxidant indices with dietary biochar supplementation. This indicates that birds fed dietary biochar had minimal exposure to lipid peroxidation, likely due to improved antioxidant defense capacity. Similarly, biochar exhibited the ability to enhance both non-enzymatic and enzymatic antioxidant systems (such as SOD, catalase, and glutathione peroxidase), leading to a reduction in toxicity (Hasanuzzaman et al. 2021). El-Ghalid et al. (2022) reported a significant increase in serum antioxidant indices, which can be attributed to dietary biochar’s ability to inhibit free radicals and maintain normal enzymatic activity. Overall, biochar supplementation enhances the antioxidant status of broiler chickens by exerting direct antioxidant benefits, improving mineral retention, reducing oxidative stress, and supporting gut health.

Digestive enzyme activity represent an important factor influencing the digestive ability of birds, providing the driving force for the digestive system in the body. An increase in this activity may lead to improved feed efficiency and growth performance. The digestive enzyme activity was enhanced with increasing dietary biochar levels. Zhang et al. (2021) observed a significant increase in lipase, amylase, and trypsin levels by supplementing 1% probiotics in the drinking water for broilers. However, there is limited literature regarding the impact of biochar on digestive enzyme activity in broilers. It is worth noting that dietary biochar may function similarly to probiotics, enhancing digestive enzyme activity. Thus, biochar improves digestive enzyme activity by providing surfaces for enzyme attachment, optimizing gut pH, and enhancing mineral availability.

Biochar supplementation can enhance gut health in birds by providing surfaces for beneficial bacteria and by modulating gut pH to create a favorable environment for beneficial microbes while inhibiting harmful ones. Additionally, biochar improves mineral availability, mitigates oxidative stress, and strengthens intestinal integrity. Collectively, these effects promote a healthier microbial environment by increasing the presence of beneficial bacteria and reducing harmful ones. Biochar also promotes an oxidized and reduced environment, leading to decreased anaerobic activity. Prasai et al. (2016) illuminated that using biochar, zeolite, and bentonite can selectively reduce the abundance of major poultry zoonotic pathogens without compromising the diversity of the chicken microbiota or causing significant alterations in the gut microbial community. As a result, these substances are considered viable alternatives to antibiotics in poultry production. According to Goiri et al. (2021), dietary biochar at 3% concentration had no significant impact on the diversity and species of caecal bacteria, but it altered the community structure of the bacteria.

The biochar has been shown to exhibit carbohydrase enzyme-like properties, effectively decreasing the viscosity of the digesta. Thus, slower passage of feed material in the digestive tract may enhance nutrient utilization (Mumpton and Fishman 1977). The elevated viscosity negatively affects the function of digestive enzymes and the subsequent transit of nutrients prior to their absorption. As the level of dietary biochar increased, the pH of the digestive tract increased, likely due to the alkaline nature of biochar. On the other hand, according to Goiri et al. (2021), dietary supplementation with 3% biochar had no significant effect on intestinal pH.

The dietary biochar supplementation had a positive impact on various carcass characteristics of broilers across treatments. This improvement could be attributed to the influence of biochar on the birds’ BWG, resulting in increased carcass weight and related traits. These findings are in line with Kutlu et al. (2001), Majewska et al. (2011), Jiya et al. (2014), and El-Ghalid et al. (2022). Regarding gizzard percentage, birds fed dietary biochar exhibited higher values compared to the control group, the same result was clarified by Kutlu et al. (2001) and El-Ghalid et al. (2022). Activated dietary charcoal could be the reason for the increased gizzard size (Jiya et al. 2014).

Finally, through the current study, several mechanisms explain how biochar improves growth performance and physiological status: first, by enhancing the antioxidant system through its detoxification effects; second, through positive effect on digestive enzyme activity, and digesta viscosity and pH, thereby improving feed utilization; and third, by reducing pathogenic microbiota.

## Conclusion

Based on the present findings, dietary supplementation with biochar at levels of 1.5% to 3.0% acted as a functional feed additive that positively influenced various aspects of broiler health, performance, and nutrient utilization, blood parameters, antioxidant capacity, and intestinal microbiota, thereby highlighting its potential as a valuable dietary additive for improving poultry production.

## Data Availability

The data that support the findings of this study are available on request from the corresponding author.

## Declarations

### Animal welfare

This study was approved by Fayoum University Institutional Animal Care and Use Committee (FU-IACUC), Egypt (Code No. of approval: AEC2342).
